# First description of the male of *Cryptothele verrucosa* L. Koch, 1872 (Araneae), the type species of the genus

**DOI:** 10.3897/zookeys.351.6255

**Published:** 2013-11-15

**Authors:** Yuri M. Marusik, Mikhail M. Omelko

**Affiliations:** 1 Institute for Biological Problems of the North, RAS, Portovaya Str. 18, Magadan 685000, Russia; 2 Zoological Museum, University of Turku, FI-20014 Turku, Finland; 3 Gornotaezhnaya Station FEB RAS, Gornotaezhnoe Vil., Ussuriyski Dist., Primorski krai 692533 Russia; 4 Far Eastern Federal University, Sukhanova, 8, Vladivostok 690950 Russia

**Keywords:** Spider, Zodariidae, Cryptothelidae, Fiji

## Abstract

The male of *Cryptothele verrucosa* L. Koch, 1872, the type species of *Cryptothele* L. Koch, 1872, known from Fiji and Samoa, is described for the first time. It is compared with the male of *C. alluaudi* Simon, 1893, the single properly described species of the genus.

## Introduction

*Cryptothele* L. Koch, 1872 is a small genus of litter dwelling spiders, the bodies of which are covered with dirt. Up to now eight species and two subspecies are known in the genus. The genus occurs from Seychelles to Fiji and Samoa. Family placement of *Cryptothele* remains uncertain. Originally the genus was placed in a separate family Cryptothelidae L. Koch, 1872. Soon after, Simon (1890) downgraded this group to a subfamily level and placed it in Zodariidae Thorell, 1881, although Cryptothelidae had formal priority. This group was returned to family status by Davies (1985), but then Wunderlich (2004) reduced its status back to a subfamily of Zodariidae. Currently (Platnick 2013) the younger name, Zodariidae, is amply protected by usage.

Of ten species and subspecies described to date, only one species, *Cryptothele alluaudi* Simon, 1893, is relatively well described and its somatic morphology and the conformation of copulatory organs studied (Saaristo 2010; Marusik and Omelko 2012). Five of ten species and subspecies are known from females only; one species, *Cryptothele cristata* Simon, 1884, with an unknown type locality, is described from a juvenile, and the description of *Cryptothele collina* Pocock, 1901 is based on specimens for which there are no indication of sex or stage (Platnick 2013).

Working with collections of the Zoological Museum, University of Turku we found a single male from Fiji identified by Pekka Lehtinen as *Cryptothele verrucosa* L. Koch, 1872. *Cryptothele verrucosa* is the type species of the genus and known on the basis of female sex and only from Fiji and Samoa. Since its description it has never been considered in taxonomic papers (*cf.* Platnick 2013). Although the species is known from females only, and original description is rather poor it is reasonable to conclude that the identification made by Lehtinen is correct, because all species of the genus have allopatric distribution (*cf*. Fig. 10, Marusik and Omelko 2012). Therefore, the purpose of this paper is to provide the first description of male of *Cryptothele verrucosa*.

## Material and methods

Specimens were photographed using an Olympus Camedia E-520 camera attached to an Olympus SZX16 stereomicroscope. The images were montaged using “CombineZP” image stacking software. Photographs were taken in dishes of different sizes with paraffin in the bottom. Different sized holes were made in the paraffin to keep the specimens in the correct position. The studied material is kept in the Zoological Museum, University of Turku (ZMUT). All measurements are in mm.

## Taxonomy

### Cryptothele verrucosa

L. Koch, 1872

http://species-id.net/wiki/Cryptothele_verrucosa

Figs 1–3 , 6–11

Cryptothele verrucosa L. Koch, 1872: 240, pl. 20, f. 2 (D*♀*).

#### Material examined.

FIJI: 1*♂* (ZMUT AA 5.828), Viti Levu, Suva rain forest, 26.05.1973 (J.M. Ackerman).

#### Diagnosis.

*Cryptothele verrucosa* differs from *Cryptothele alluaudi*, the only properly described species in the genus, by lack of carapace pattern, much more heavy camouflage of dirt that covers whole body, straight row of posterior eyes, anterior lateral eyes spaced by one diameter (1/2 of diameter in *Cryptothele alluaudi*), shape of tibial apophysis (*cf*. Figs 11, 12), short and broad embolus with two processes (long and filamentous in *Cryptothele alluaudi*, Fig. 12).

**Figures 1–5.**
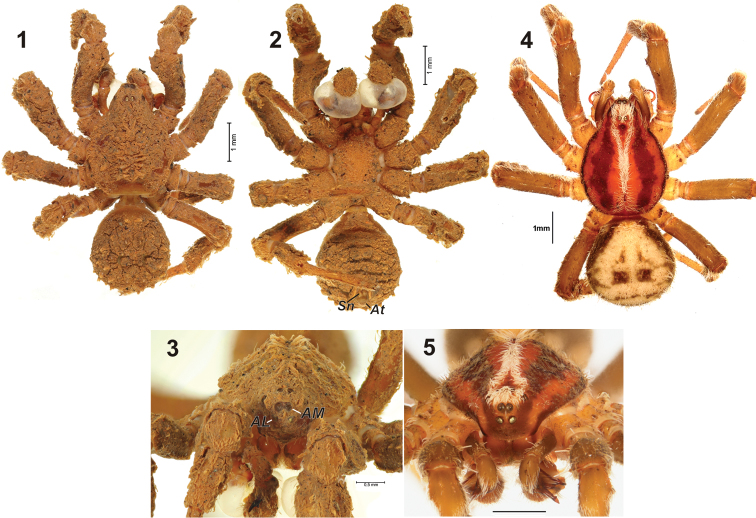
General appearance of males of *Cryptothele verrucosa* (**1–3**) and *Cryptothele alluaudi* (**4–5**). **1, 4** dorsal **2** ventral **3, 5** frontal **4–5** after Marusik and Omelko (2012). Abbreviations: ***AL*** anterior lateral eye; ***AM*** anterior median eye; ***At*** anal tubercle, ***Sn*** spinneret.

**Figures 6–12.**
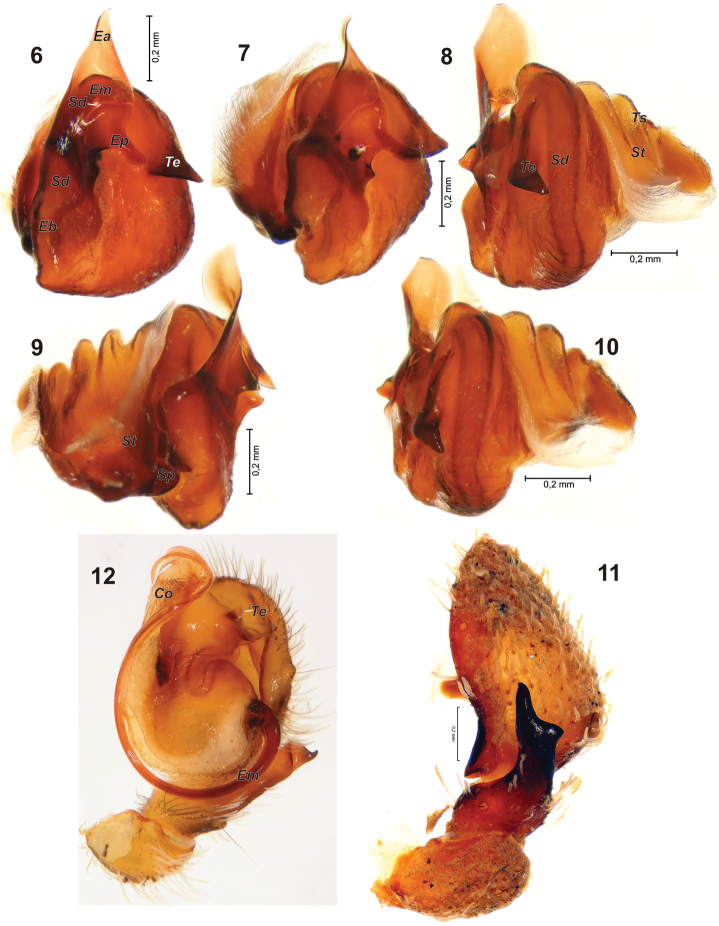
Male palp of *Cryptothele verrucosa* (**6–11**) and *Cryptothele alluaudi* (**12**). **6** bulbus, ventral **7** bulbus, ventro-prolateral **8, 10** bulbus, retrolateral **9** bulbus, prolateral **11** palp with removed bulbus, retrolateral **12** palp, ventral (after Marusik and Omelko (2012)). Abbreviations: ***Co*** conductor; ***Ea*** terminal process of embolus; ***Eb*** embolus base; ***Em*** embolus; ***Ep*** posterior process of embolus; ***Sd*** seminal duct; ***Sp*** subtegular process; ***St*** subtegulum; ***Te*** triangle extesion of tegulum; ***Ts*** threads of subtegulum.

#### Description.

Measurements. Total length 5.5, carapace 3.0 long, 2.28 wide. AME 0.14, AME-AME 0.15. Position of metatarsal trichobothria IV 0.9.

Whole body, including sternum and venter of abdomen covered by comouflaging dirt (Figs 1–2). Carapace brown, without pattern, with two rows of hairs aside of median line, these rows are visible after removing the comouflaging dirt. AME spaced by one diameter (Fig. 3), anterior eyes form inverted trapezium (ALE row wider than AME row), posterior eye row almost straight, cephalic area with pit behind posterior median eyes. Leg subeaqual in length, formula 1423. Legs heavily built, with thick femur–tibia and twice as thin metatasrus–tarsus, border between tarsus and metatarsus poorly visible, metatarsi with terminal trichobothria.

Length of legs and leg joints.

Abdomen oval, with two spinnerets (*Sn*) about the size of anal tubercle (*At*).

Palp as shown in Figs 6–11. The single specimen examined has both palps expanded. Tibia (Fig. 11) with long retrolateral apophysis tapering in terminal 1/3, dorsal side of apophysis with shallow blunt outgrowth, retrolateral side of tibia with trichobothrium in proximal part; cymbium oval, with trichobothrium on retrolateral side. Subtegulum (*St*) (Figs 8–10) large (as long as tegulum in lateral view), cone-shaped with three threads (*Ts*); prolaterally with process (*Sp*) directed to notch of embolic base. Tegulum nearly oval with triangle extesion (*Te*) in terminal part (Figs 6, 8, 10). Embolus (*Em*) broad, longer than tegulum, heavily built in the base (*Eb*), terminal part lamellated with two processes: digitiform posterior process (*Ep*) and triangle shape terminal process (*Ea*), seminal duct (Sd) broad and heavily sclerotised in the base of embolus, and very fine in lamellar part of embolus.

#### Comments.

Thanks to the discovery of the male of *Cryptothele verrucosa* (the easternmost species of the genus) it has became possible to compare it with the westernmost species, *Cryptothele alluaudi*. General appearance of the two species is rather similar (Figs 1–5). They differ in amount of camouflage cover which is almost absent in *Cryptothele alluaudi*,but *Cryptothele verrucosa* is covered heavily on dorsal and ventral sides. Both male and female of *Cryptothele alluaudi* have a distinct pattern on the carapace. Such a pattern is absent in the studied male of *Cryptothele verrucosa* (we have removed the camouflage cover); it is also absent in the conspecific female, judging from L. Koch’s description. Male of *Cryptothele alluaudi* has relatively longer and thinner legs (cf. Figs 1 and 4) and less spaced anterior lateral eyes (Figs 3 and 5). The posterior eye row is straight in *Cryptothele verrucosa* (Fig. 1) and recurved in *Cryptothele alluaudi* (Fig. 4). Male palps in two species are strikingly different. *Cryptothele alluaudi* has a long filamentous spiraled embolus and conductor (*Co*) (Fig. 12). In *Cryptothele verrucosa* the embolus is flat and broad. Extension of tegulum in *Cryptothele alluaudi* is weakly sclerotized and has subparallel margins, while in *Cryptothele verrucosa* it is triangle–shaped and strongly sclerotized.

In the collection of the Zoological Museum, University of Turku we had the opportunity to examine males of two unidentified species of *Cryptothele*, one from Thailand (which seems new to science) and another from Indonesia (probably *Cryptothele sundaica* Thorell, 1890). Males of both species have conformation of the palp similar to that in *Cryptothele alluaudi* (a thin and long embolus, a well developed conductor, etc.). This may indicate that *Cryptothele alluaudi*, *Cryptothele sundaica* and the mentioned undescribed species most likely are not congeneric with *Cryptothele verrucosa* and the genus could be split in the future into two separate genera.

#### Comparative material examined.

*Cryptothele* sp. (presumably new) 1*♂* (ZMUT: AA 5.812), Thailand, Chanthaburi Pr., Kho Yai N.P., Wang Chum Pee, rain forest, 27.10–22.11.1976 (P.Lehtinen) covered with dirt, even cymbium, but RTA like in *Cryptothele alluaudi*, embolus and conductor long.

*Cryptothele sundaica*?: 2*♂* 1*♀* (ZMUT: AA 5.806), Indonesia, Sumatera Barat, Paykumbuh d., Lubu Bangku, low jungle, 7.12.1980 (P.T.Lehtinen).

## Supplementary Material

XML Treatment for Cryptothele verrucosa
